# Causes and consequences of quack medicine in health care: a scoping review of global experience

**DOI:** 10.1186/s12913-023-10520-9

**Published:** 2024-01-11

**Authors:** Ali Amir-Azodi, Mohammad Setayesh, Mohammad Bazyar, Mina Ansari, Vahid Yazdi-Feyzabadi

**Affiliations:** 1 Health Services Management Research Center, Institute for Futures Studies in Health, Kerman University of Medical Sciences, Kerman, Iran; 2 Department of Traditional Medicine, Faculty of Persian Medicine, Kerman University of Medical Sciences, Kerman, Iran; 3 Health Management and Economics Department, Faculty of Health, Ilam University of Medical Sciences, Ilam, Iran; 4 Faculty of Management and Medical Information, Kerman University of Medical Sciences, Kerman, Iran

**Keywords:** Quack medicine, Quackery in health care, Counterfeit medicine, Health service provision system

## Abstract

**Background:**

The field of health has been facing challenges with fraudulent practices and the prevalence of “quack medicine”. Many cases have given rise to this issue. Therefore, this study aims to comprehensively investigate and categorize the causes and consequences of quack medicine in the healthcare.

**Methods:**

A scoping review, using the 5 stages of Arksey and O’Malley’s framework, was conducted to retrieve and analyze the literature. International databases including the PubMed, Scopus, Embase and Web of Science and also national Iranian databases were searched to find peer reviewed published literature in English and Persian languages. Grey literature was also included. Meta-Synthesis was applied to analyze the findings through an inductive approach.

**Results:**

Out of 3794 initially identified studies, 30 were selected for this study. Based on the findings of this research, the causes of quackery in the health were divided into six categories: political, economic, socio-cultural, technical-organizational, legal and psychological. Additionally, the consequences of this issue were classified into three categories: health, economic and social. Economic and social factors were found to have a more significant impact on the prevalence of quackery in the health sector. Legal and technical-organizational factors played a crucial role in facilitating fraudulent practices, resulting in severe health consequences.

**Conclusion:**

It is evident that governing bodies and health systems must prioritize addressing economic and social factors in combating quackery in the health sector. Special attention should be paid to the issue of cultural development and community education to strengthen the mechanisms that lead to the society access to standard affordable services. Efforts should be made also to improve the efficiency of legislation, implementation and evaluation systems to effectively tackle this issue.

## Background

Issues such as quackery and charlatanism have long been a concern for human civilizations and communities since the development of health service delivery structures and processes. While the forms and severity of these issues may vary across nations, it can be argued that all health systems are susceptible to some level of quackery [1]. In the health system, particularly in developing countries, a phenomenon known as “Quack Medicine” has been a persistent problem, causing harm in various branches of health care services. Quackery refers to unproven or fraudulent medical practices that there is no scientifically plausible rationale behind them. Furthermore, someone who does not have professional qualification, formal registration from a legitimated institution, or required knowledge of a particular branch of medicine but practices in the field of medicine, is called quack [2–4]. So quack medicine refers to the fraudulent practice of quacks in the medical field claiming to possess the ability and experience to diagnose and treat diseases, and pretending that the medicine or treatment they provide are effective, generally for personal and financial gain [5]. A study from India defines the following individuals as quacks as well: practitioners of local types of medicine such as Indian Medicine Ayurvedic and Homeopathy who practice modern practice although they are not allowed to do so and those who engage in any type of medicine which have not been recognized by law [6]. Other examples of this phenomenon include billing for services not provided, substituting substandard products for standard ones, taking unnecessary steps to get more reimbursement, and prescribing unnecessary medications for financial gain instead of addressing medical needs [7].

In a historical survey conducted by the American Medical Ethics Association in 2000, it was found that in 1775, only 400 out of every 3,000 individuals claiming to be doctors had legitimate medical degrees from accredited universities and schools. Also, the vast geographical size of this country and the dispersion of experienced doctors led to the public seeking to refer to quack doctors. In the 19th and 20th centuries, these charlatans utilized pseudo-scientific terms and advanced equipment and technologies to deceive people, blurring the line between a legitimate doctor and a quack [8]. In 2020, the Independent newspaper reported on this issue in Bangladesh, revealing that 75% of quack doctors were prescribing inappropriate medications to patients, with 7% prescribing drugs that were completely harmful and dangerous [9].

The seriousness of this problem led the World Health Organization (WHO) to hold an international conference in Rome in 2006 entitled “Combating Counterfeit Medicines: Establishing Effective International Cooperation”. As a result, a framework document was created to address and control and combat this issue [10]. The WHO has also established a mechanism for member countries to collaborate and take action against substandard and fake medical products. Additionally, a global monitoring and surveillance system has been implemented to encourage countries to report substandard and fake medical cases in a systematic structure, aiding in a more accurate and reliable assessment of the problem [11].

Numerous studies from different parts of the world have highlighted the harmful effects of quack medicine on the health and well-being of society [12–14]. A study in India, while discussing the economic and health consequences of counterfeit drugs, concluded that a multilateral approach is necessary to prevent the spread of this problem [15]. Similarly, a study in the USA identified quackery in healthcare as a complex issue involving economic, moral, cultural, social and educational factors that reinforce one another [16].

Recent events, such as the outbreak of the coronavirus pandemic and the lack of a specific treatment for it, have further emphasized the need to address the issue of quack medicine in the health system. For example, the US Food and Drug Administration Regulatory Affairs Commission announced that since the onset of the pandemic, over 700 fake and unproven medical products claiming to treat the virus have been identified and dealt with [17].

In order to effectively combat and prevent charlatanism, it is crucial to understand the underlying causes and complexities of this issue. Without addressing the root causes and consequences, the issue cannot be properly rectified within the healthcare system. This understanding will help scholars and policy makers recognize the importance of quack medicine and take it seriously. Recognizing the underlying causes and various factors that can lead to the prevalence of medical quackery is the first step in devising purposeful policies to tackle the problem. The authors believe that quack medicine is a multidimensional phenomenon affected by many different factors which in turn requires multifaceted educational, legal, and structural programs and application of complicated strategies in various areas of society simultaneously to prevent, early detect, and provide prompt and deterrent response to it [16]. Therefore, this study aims to comprehensively investigate, identify, and classify the causes and consequences of charlatanism in the health service provision system, given the significance of this issue and the sporadic attention it has received in previous research.

## Methods

A scoping review was conducted using the 5 stages of Arksey and O’Malley’s framework to identify and classify the main reasons for the occurrence of quack medicine around the world and its impact on the healthcare system. Scoping reviews are useful for examining emerging evidence when it is not still possible to pose more specific questions appropriate to addressed by a more precise systematic review. Scoping reviews are more applicable when the aim of study is to identify and map the available evidence or identify and classify the main characteristics or factors related to a topic [18, 19].

### Stage 1: Identifying the initial research questions

The main questions addressed in this review were: (1) What are the main reasons for the prevalence of quack medicine in the healthcare? (2) and What are the consequences of quack medicine on the healthcare?

### Stage 2: Identifying relevant studies

To extract relevant documents, a comprehensive search was conducted on international databases including the PubMed, Scopus, Embase and Web of Science to find peer reviewed literature published in English. Appendix 1 shows the search strategies used to extract relevant studies. Grey literature was also searched on international organization websites such as WHO, Food and Drug Administration (FDA) and International Transparency Organization. National Iranian databases were also searched for relevant literature in Persian. These databases included SID, Magiran and IranDoc. Furthermore, reference lists of selected documents were also scanned for additional relevant articles. The details of all the selected studies were saved in EndNote X7 software, which can be used to find duplication in extracted studies.

### Stage 3: Study selection

A total of 3794 documents were retrieved from the initial search, of which 2477 were removed due to duplication. Then two of authors reviewed the title of 1317 remained articles and 852 documents further removed as they were not found to address the topic directly. In the next step, after the first screening, the entire texts of 465 articles were checked based on the exclusion criteria established for the research, and 435 were rejected. Eventually 30 papers were included for the purpose of the study, and the causes and effects of charlatanism in the field of health were used to explain the results.

**Fig. 1:**
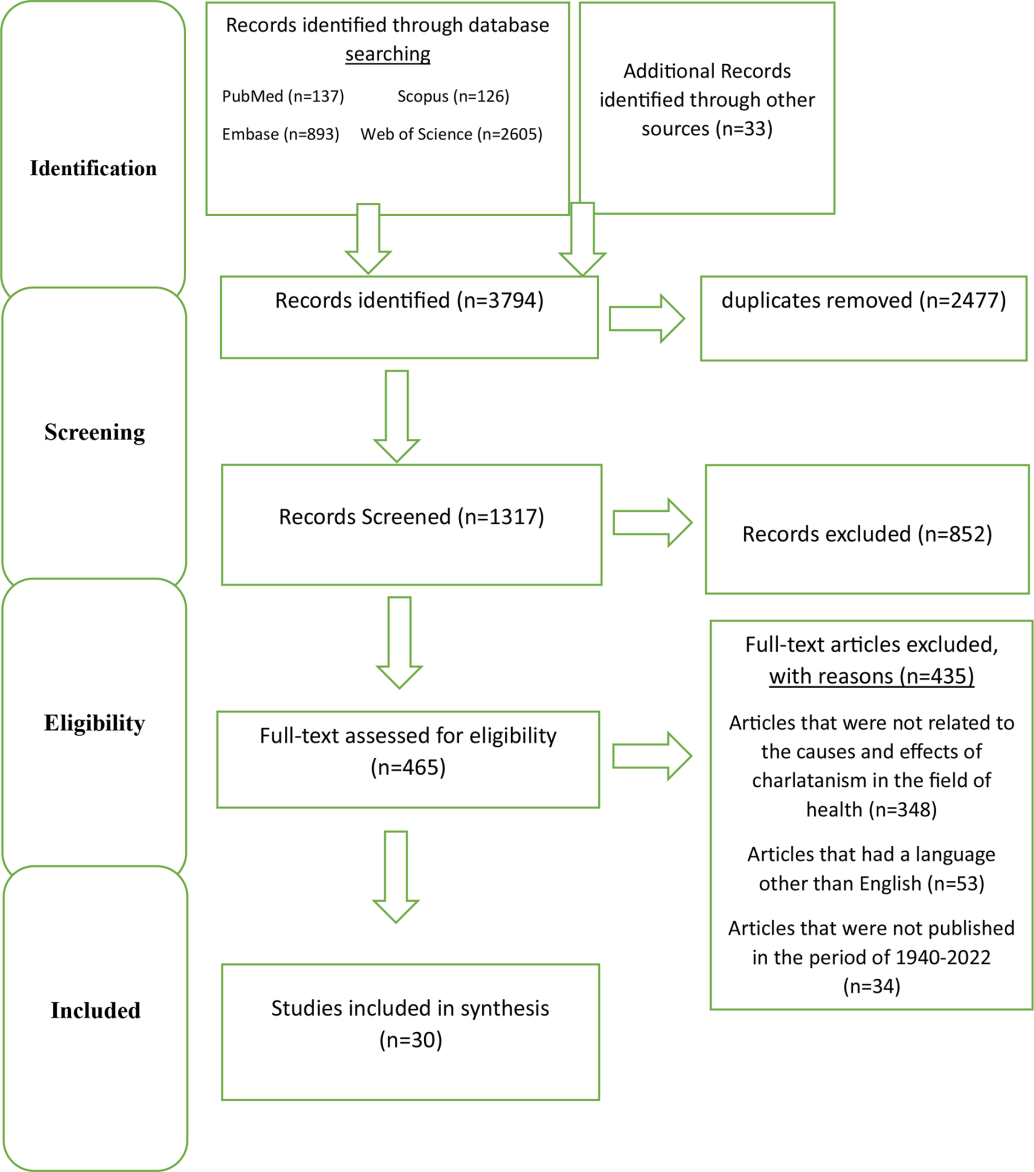
PRISMA flowchart for selection of articles

The process of study selection is shown in Fig. 1 (PRISMA flowchart).

### Inclusion and exclusion criteria

The present study has included articles that their full-text were available, written in English, published between 1940 and 2022, and addressed the causes and consequences of quackery in the field of health. The studies on the history of quack medicine or unrelated to health care provision were excluded.

### Stage 4: Data charting

A worksheet was created in Excel to extract information for the selected articles, including the first author, the year of the publication, title, country, type of study and design of the study (see Table 1). The similar information was extracted for grey literature (see Table 2). In addition, the frequency of studies included in the research by date, type and design is shown in Table 3.

### Stage 5: Collating, summarizing and reporting the results

Meta-Synthesis was applied to analyze the findings through an inductive method. The full text of all finalized documents were studied carefully. Findings of the studies, the reasons and the consequences of quack medicine, were summarized in two separate tables. These summaries were then grouped into broader categories based on the similarities between them. During the final phase, the research team engaged in a thorough discussion regarding the initial categorization of reasons and consequences. Amendments were made as needed including paraphrasing headings, modifying classifications or transferring sub-categories. This iterative process continued until consensus was achieved. Tables 4 and 5 show the final categorization for factors contributing to quack medicine and its impacts on healthcare respectively.

**Table 1:** List of the included studies

First Author	Date	Title	Country	Type	Study Design
Shrivastava	2014	Public health measures to fight counterfeit medicine market	India	Letter to Editor	Qualitative
Jarvis	1999	Quackery: The national council against health fraud perspective	USA	Review	Review
Kalb	1999	Health care fraud and abuse	USA	Review	Review
Miller	2013	Exposing medical fraud: “one of the last taboos in society”	Canada	Note	Qualitative
Stelfox	2003	An analysis of one potential form of health care fraud in Canada	Canada	Letter to Editor	Qualitative
Avery	1996	Congress focuses on health care fraud and abuse	USA	Commentary	Commentary
Dear	2007	Disease mongering - A challenge for everyone involved in healthcare	United Kingdom	Note	Qualitative
Cowart	1988	Health fraud’s toll: lost hopes, misspent billions	USA	Opinion	Qualitative
Johnson	1989	The health fraud battle. Education is the best defense	USA	Original Article	Qualitative
Hosseini	2011	Counterfeit medicines: Report of a cross-sectional retrospective study in Iran	Iran	Original Article	Cross-Sectional
Mc Cullough	2015	An Interview with Deputy Chief, Health Care Fraud Unit at the U.S. Attorney’s Office	USA	Interview	Qualitative
Lohsiriwat	2007	Fraud and deceit in published medical research	Thailand	Review	Review
Sparrow	1996	Health care fraud control: understanding the challenge	USA	Original Article	Qualitative
Jarvis	1992	Quackery: a national scandal	USA	Review	Review
Bernard	1965	Why People Become the Victims of Medical Quackery	USA	Opinion	Qualitative
Worrall	1990	Detecting health fraud in the field of learning disabilities	USA	Opinion	Qualitative
Akunyili	2004	Risk of medicines: Counterfeit drugs	Nigeria	Original Article	Qualitative
Nsimba	2008	Problems associated with substandard and counterfeit drugs in developing countries	Nigeria	Review	Review
Price	2009	Health care fraud: Physicians as white-collar criminals?	USA	Editorial	Qualitative
Miller	2013	Medical fraud north of the 49th	Canada	Note	Qualitative
Widder	2015	The appeal of medical quackery: a rhetorical analysis	USA	Commentary	Commentary
Iroegbulem	2020	Disease Mongering: How Sickness Sells	USA	Commentary	Commentary
Berthelot	2019	The negative Hawthorne effect: Explaining pain overexpression	France	Original Article	Qualitative

**Table 2:** List of the included gray literatures

First Author	Date	Title	Publisher
Bagozzi	2003	World Health Organization steps up action against substandard and counterfeit medicines	World Health Organization
Lindmeier	2017	Seventieth World Health Assembly update, 29 May 2017	WHO
Dovlo	2016	Dr Moeti proposes actions to address fake medical products	WHO
Kasilo	2014	We must prevent the production, marketing and use of unsafe medical products, says Dr Sambo, WHO Regional Director for Africa	WHO
Trapsida	2010	Interventions for prevention and control of substandard/spurious/falsely labelled/falsified and/or counterfeit medical products in the WHO African Region	WHO
-	2013	Know the Risks	U.S. Food and Drug Administration
-	2020	About Be Safe Rx	U.S. Food and Drug Administration

Among the retrieved documents, the highest number of articles published within the period of 2011–2022 (13 out of 30). The number of included studies by type is 5 original studies, 5 review studies and 20 studies from other types. Furthermore, almost half of the selected studies were qualitative, followed by five review, three commentary, one cross-sectional and seven studies had no specific design. The frequency percentage of studies according to the mentioned characteristics is shown in the Table 3.

**Table 3:** A summary of characteristics of included studies

Variable	Frequency (%)
**Time/date**	
Before 2000	9 (30.0)
2001–2010	8 (26.7)
2011–2022	13 (43.3)
**Study type**	
Original	5 (16.7)
Review	5 (16.7)
Other types	20 (66.6)
**Study design**	
Qualitative	14 (46.7)
Review	5 (16.7)
Cross-Sectional	1 (3.3)
No design	10 (33.3)

## Results

The results of the scoping review are organized into two main themes: the causes and effects of charlatanism and quack medicine in the healthcare system.

### Causes of quack medicine

Based on the results of this study, the causes of quack medicine in the health system are divided into six categories: political, economic, sociocultural, technical-organizational, legal and psychological factors. These categories are further explained as follows. Table 4 shows the categories and sub-categories of causes in brief.

### Political factors

This category encompasses factors related to the involvement of political authority or governments in certain industries and systems. The following reasons have been identified based on the results of this study:

#### Approaches influenced by political ideologies

In many countries, the prevalence of charlatanism can be attributed to policies such as international free trade, rooted in ideologies such as liberalism and neoliberalism. This can lead to charlatanism in various fields, particularly in healthcare. The most important approaches include Commercialism, Consumerism and Professionalism [20, 21]. Further explanations are provided as follows to understand how these approaches might facilitate or prohibit quack medicine.

#### Commercialism

This approach prioritizes increasing a nation’s wealth by any means, even if this involves promoting fraudulent practices. Accordingly, the government not only should not prevent quack medicine but also not take action to take care of those who may be affected negatively by fraudulent practices [21].

#### Consumerism

Consumer protection law questioned the “caveat emptor principle” which says the person who buys something is responsible for checking that it is not broken, damaged or counterfeit. The caveat emptor principle is no longer appropriate in healthcare system where there is asymmetry information and patients can be easily deceived by quackery health care providers. Consumer protection law is to protect ordinary people especially those from disadvantageous groups and with limited access to valid and reliable health information sources against charlatans [21].

#### Professionalism (competition versus cooperation)

It is said that marketplace competition in healthcare system is undermining the scientific aspects of medicine where all actors cooperate with each other to keep people healthy. This traditional view was changed fundamentally when the legislators lifted the prohibitions on advertising for associations and professions related to medicine and health care. The reason behind it was based on this belief that free market competition forces providers to keep prices low and that the health care industry needed to control its prices. However it should be noted that although health care system is a business, all aspects of patient care must be done according to the professional medical ethics. Emphasizing on competition and market principles can encourage incidence of quackery medicine in the absence of regulatory arrangements and observatory mechanisms [21].

#### Lack of political will [20]

In some countries, there may be a lack of willpower, determination and effort among political leaders to deal with and prevent fraud and charlatanism in various fields, especially in the health system. This can be due to conflict of interests, corruption network, or insufficient infrastructure and resources, such as financial capacity and human resources.

### Economic factors

The causes for charlatanism can also be attributed to growth or recession of the economy, production, distribution and consumption of goods and services as well as the financial resources of individuals and society. The following reasons have been identified based on the results of this study:

#### Weak economic condition and poverty in society [20, 22]

One of the key factors that contributes to the prevalence of charlatanism is the poverty and poor economic condition of society [20]. This factor, combined with financial incentives, creates a fertile ground for quackery and charlatanism in the health system.

#### Increasing cost of standard healthcare [23]

When the price of conventional medicines and health services rise, creating a price difference between these and alternatives products and services, there is a greater incentive for customers to seek out non-standard health services and services that are not scientifically validated. Studies have shown that low prices are a major motivation for consumers, and some may even turn to unregulated and counterfeit products and services in the healthcare industry [24].

#### Greater demand than supply of drugs [20]

Shortage of standard drugs can contribute to the issue of counterfeit medications, leading to fraudulent practices and potentially causing serious health complications or even death. In such situations, the price of a legitimate medications may increase due to a black market created by high demand and low supply, providing opportunities for profiteers to make money through fraudulent means [24].

#### Economic recession of countries

During periods of economic recession, the costs of health care fraud tend to increase, according to WHO [25]. This is because, patients may delay non-urgent medical treatments, putting licensed providers under financial strain. As a result, some providers may resort to fraudulent practices to generate more income, ultimately contributing to the overall costs of quack medicine.

#### High cost required to deal with quackery medicine

In some cases, policy makers may choose to tolerate small levels of unproven medical practices if the cost of prosecuting and correcting the situation outweigh the financial benefits. This can lead to a cycle of continued fraud and a lack of effective interventions to address the issue [26, 27].

#### Disease mongering

This phenomenon is the modern form of “medicalization” and refers to the expansion of the boundaries of treatable diseases in order to expand the market for people who benefit from the treatment [28, 29]. To increase the market for pharmacotherapy, risk factors are defined as a disease or change in diagnostic cut-off points to justify the necessity of taking treatment and encouraging people to demand them [28]. This phenomenon is commonly used by pharmaceutical companies, medical device manufacturers, insurance companies and even some doctors and patient groups, and it has become a major concern [29]. While it may have some benefits for public health, it can also be misleading and costly for patients when risk factors are falsely portrayed as easy and necessary to treat [28].

### Sociocultural factors

Some of the causes for charlatanism are rooted in social and cultural context. For instance, the norms, customs, challenges, characteristics and values of a population or society can also contribute to the prevalence of charlatanism. Some of the key findings in this regard include:

#### Illiteracy, low literacy levels and lack of awareness

In the field of public health, ignorance among individuals can leads to significant problems [24]. Uninformed individuals are often the victims of quackery and scams [30]. One of the main causes of practicing quackery in health system is a lack of knowledge about the consequences of counterfeit medicine among the general population, medical experts, and pharmacists [20].

#### Deceptive advertisements [30, 31]

Print advertising, commercial television shows, and other media can deceive consumers [30]. For example, in a research conducted in Iran, findings revealed that people had easy access to satellite TV and the Internet, allowing for direct consumer interaction with medication marketing and services. This has a significant impact on Iranians’ lifestyle choices regarding drug use and their expose to fake medical products and services [32].

### Technical-organizational factors

Some of the causes for quackery are related to the methods, structures, goals and mechanisms of the health service provision system, as well as the issues related to the existing technologies in this field. The following are key findings in this regard:

#### Reluctance of health authorities and institutions to publicize fraud cases [20]

Medical product manufacturers and pharmaceutical companies may be hesitate to report fake and counterfeit products, as they afraid that consumers might lose faith in the reliability and the safety of their goods. On the other hand, organizations that are responsible for supervising health care facilities and ensuring standard health services and products, may not be willing to publicize the news about the fraud and corruption in health system as they believe it may lead to misjudgment among people regarding their performance.

#### Online pharmacies [20, 24, 33]

Online pharmacies are a primary source of fake medicine [24]. Most of online pharmacies lack sufficient security measures to protect customers’ personal and financial information, and some of them may intentionally misuse customer’s data [34]. These pharmacies allow people to purchase drugs without a doctor’s prescription. They may also offer discounts or cheap prices that may seem legitimate. They may also send spam emails offering cheap drugs, and are located outside that country without proper licensing to provide services within the country [34]. In 2013, the National Association of American Pharmaceutical Trade Boards found that 97% of the internet pharmacies violated local, state, and federal regulations as well as business standards [24] .

#### Multiple payers and providers

Multiple payers with separated and fragmented data banks, the vast range of provider reimbursement systems and the diversity of providers result in a complicated structure that makes it challenging to identify instances of fraud and abuse in the healthcare system. It is much easier to analyze the behavior of all kinds of providers and combat quackery in a centralized health system where all information of each provider and actor are pooled in a single data center [27].

#### Fraud and deception in health research

Clinical trials and epidemiological studies are susceptible to various forms of fraud and deception, such as falsification and distortion of data, deceptive reporting of results, deceptive design or analysis, discarding negative results and selective reporting of positive results [35].

#### Involvement of secondary wholesales

While primary, large, and regional wholesalers have direct communication with drug manufacturers and are less likely to deal with counterfeit drugs, secondary wholesalers do not have this direct link and may engage in repackaging drugs, providing opportunities for fake products and counterfeit drugs to enter the market [24].

#### Operational obstacles in combating quackery

Controlling fraud as a whole in health system is a dynamic not a static game, that is those engaging in health fraud are always looking for adaptation and devising new creative strategies. So the current satisfactory solutions to control health fraud become old and ineffective very soon. It requires “continuous assessment of emerging fraud trends and constant, rapid, revision of controls”. This is also true in addressing quack medicine [36].

### Legal factors

A part of quackery in the healthcare rises from deficiencies existing in the laws and judicial system. Following legal factors are among the main contributors:

#### Absence or inadequacy of regulatory mechanisms [20]

A strong regulatory authority is crucial in ensuring compliance with laws and guidelines in the healthcare sector. Without proper oversight, fraudulent activities can thrive.

#### Legal loopholes [20]

The absence or inadequacy of laws related to fake health services and products creates opportunities for charlatans and profiteers to exploit the system.

#### Ineffective implementation of existing laws [37]

While laws may exist, their effectiveness depends on proper and strict implementation. More efforts and measures must be taken to implement the existing laws. Inadequate enforcement of laws and approval of pseudo-medicine can result in people receiving improper care [21].

#### Non-deterrent criminal penalties

The prevalence of quackery in the healthcare system is greatly influenced by the effectiveness of legal sanctions in deterring these illegal and harmful actions. In countries where harsh and deterrent penalties are not enforced, quacks and fraudsters may be encouraged to continue their fraudulent practices. This is evident in Iran, where existing laws do not adequately punish fraudulent providers and distributors of counterfeit drugs, despite the potential for these drugs to cause death. As a result, these individuals may not face appropriate consequences for their actions, leading to a perpetuation of quackery in the healthcare system [32].

### Psychological factors

In addition to the aforementioned economic and legal factors, psychological factors also play a significant role in the prevalence of quack medicine in the healthcare system. These include:

#### Vulnerability of patients

One of the main emotional reasons for falling prey to quackery is fear, particularly fear of death and disability, and the corresponding desire for survival and good health [31]. Vulnerable individuals are more likely to be enticed by the false promises of charlatans, especially in today’s fast-paced and impersonal medical practices [16]. This vulnerability is further amplified in patients with serious, chronic or painful condition who may be desperate for relief [30]. Additionally, patients with learning disorders are particularly susceptible to fraudulent treatments [38].

#### Disappointed healthcare providers

On the other hand, some providers may resort to quackery due to their frustration with societal injustices and exclusion. They may feel marginalized and pushed down by the existing structures and systems of society. In response, they may use various prefixes to prove that these unfair discriminations have made it impossible to get what they deserve via ethical and legal ways. They may believe that traditional methods of earning money are futile, so they may turn to fraudulent practices as a means of survival [16].

**Table 4:** Causes of quack medicine in the healthcare system

Categories	Sub-categories
Political factors	Approaches influenced by political ideologies: ‣Commercialism ‣Consumerism ‣Professionalism (Competition Versus Cooperation) Lack of political will
Economic factors	Weak economic condition and poverty Increasing cost of standard healthcare Greater demand than supply of drugs Economic recession of countries High cost required to deal with quackery medicine Disease mongering
Sociocultural factors	Illiteracy, low literacy levels and lack of awareness Deceptive advertisements
Technical-Organizational Factors	Reluctance of health authorities and institutions to publicize fraud cases online pharmacies Multiple payers and providers Fraud and deception in health research Involvement of secondary wholesales Operational obstacles in combating quackery
Legal Factors	Absence or inadequacy of regulatory mechanisms Legal loopholes Ineffective implementation of existing laws Non-deterrent criminal penalties
Psychological factors	Vulnerability of Patients Disappointed healthcare providers

### Consequences of quack medicine

Based on the findings of this research, the consequences of quackery in the healthcare industry are divided into three main categories: health, economic and social. These effects are discussed in detail as follows. Table 5 shows the classification of the effects in brief.

**Table 5:** Categories and sub-categories of the consequences of quack medicine in healthcare system

Categories	Sub-categories
Health consequences	Increasing mortality Reducing medical effectiveness Preventing the achievement of treatment goals Increased risk of poisoning Adverse drug reactions Drug resistance Multiple drug interactions Negative effects caused by disease mongering
Economic consequences	Financial loss Resources waste Increased costs and burden on health care system Negative impacts on health insurance Effects caused by disease mongering
Social consequences	Undermining trust in medicine Undermining scientific activities and the credibility of the medical profession Loss of trust in health care professionals Weakening of modern medical ethics

### Health consequences

Individuals who fall victim to quackery in the healthcare field often suffer from various physical and psychological consequences that fall under the category of health effects. These include:

#### Increasing mortality [20, 22, 24, 30, 39, 40]

Mortality is the most severe consequence of quackery in the healthcare system. In the case of counterfeit drugs, not only do they lack beneficial chemicals, but they may also contain harmful substances that can lead to death [24]. Due to a lack of information and research, as well as a lack of a global coordination in combating counterfeiting, the exact global mortality rate caused by these drugs is unknown [39]. However, a study in Nigeria revealed that in 1995, over 50,000 people received vaccines imported from Niger that lacked active ingredients, resulting in the death of 2500 individuals according to the World Health Organization [41].

#### Reducing medical effectiveness [22, 24, 39]

Counterfeit medicine may not contain any effective or beneficial substances, rendering them ineffective in treating the patient’s condition. This can ultimately harm the patient’s health [24].

#### Preventing the achievement of treatment goals [31, 40]

The production, marketing and use of unsafe medical products can lead to treatment failure and even death [40]. This is because these products do not directly harm patients, but rather deny or delay effective treatment [31].

#### Increased risk of poisoning [39]

Consuming counterfeit products puts individuals at risk of poisoning, which can have long-standing impacts on their health.

#### Adverse drug reactions [39]

Taking medications may cause harmful and unwanted reactions. In the case of consuming counterfeit drugs, the risk of experiencing these kinds of reactions normally rises substantially.

#### Drug resistance

The use of unsafe and low-quality drugs can lead to drug resistance, putting the patient’s health at risk [42].

#### Multiple drug interactions [20, 31]

Taking multiple drugs simultaneously, even if they are standard, can alter their effectiveness. If these drugs are counterfeit, the consequences for the patient can be severe.

#### Negative effects caused by disease mongering

This phenomenon can lead to iatrogenic injuries. Iatrogenic injuries are special complications or diseases that occur as a result of treatment [28]. Moreover, this phenomenon can also exacerbate existing illnesses and cause anxiety, depression and nervousness in individuals [43].

### Economic consequences

Fraudulent actions in the healthcare industry have significant economic and commercial effects of individuals and society that can be irreparable. These consequences are classified as follows:

#### Financial loss [30]

Individuals who fall victim to healthcare fraud often suffer a financial loss as they spend their limited financial resources on ineffective or harmful products or services.

#### Resources waste

Counterfeit drugs not only pose a danger to patients but also to the pharmaceutical industry, health care providers, and the entire health care system. This results in a waste of economic resources [39]. Similarly, fraudulent services can lead to unnecessary costs which would be finally paid by health insurance organizations.

#### Increased costs and burden on health care system [20, 44]

All negative health consequences of quack medicine mean imposing unnecessary financial burden on the health system. It diverge the financial resources on receiving effective medical services [44]. Fraudulent services may also increase referrals to health insurance funds and put more financial pressure on the country’s insurance system.

#### Negative impacts on health insurance [44, 45]

Fraudulent activities by healthcare providers can have destructive effects on patients’ health insurance and may also put the employment of individuals at risk in the future [45]. False medical records created by providers can make it difficult for patients to obtain disability or life insurance policies later on [44]. Additionally, an inaccurate medical history can also influence treatment decisions and allow some insurance companies to deny coverage based on preexisting conditions [44].

#### Effects caused by disease mongering

Disease mongering using compelling marketing strategies tries to persuade healthy people they have a medical condition which can lead to unnecessary prescribing and increasing costs for publicly funded health service. It can also result in significant expenses by diverting funds from more cost-effective treatments [28].

### Social consequences

Fraudulent actions in healthcare industry can have significant consequences, affecting people’s attitudes and thoughts. These include:

#### Undermining trust in medicine

Counterfeit medicines can cause uncertainty and doubt about the value of the legitimate medications, leading people to turn to less effective alternatives [24]. This can erode trust not only in certain brands but also in the entire pharmaceutical industry and healthcare system [20, 46].

#### Undermining scientific activities and the credibility of the medical profession [37, 44]

Medical quackery not only harms people, but also undermines scientific activities and must be actively opposed by all scientists [37]. Quack medicine also tarnishes the credibility of the medical profession and raises questions about the ethical standards governing the practices of physicians [44].

#### Loss of trust in health care professionals [42, 46, 47]

Distribution of illegal and low quality drugs and unsafe products, as well as excessive use of non-standard, fake, falsely labeled or counterfeit products, can lead to a loss of trust in healthcare professionals, drug manufacturers, distributors and the healthcare system as a whole [42, 47].

#### Weakening of modern medical ethics

Fraudulent individuals often use manipulative tactics to convince the public, which can weaken modern medical ethics. This can have far-reaching consequences for the healthcare industry and society as a whole [48].

## Discussion

The prevalence of quackery in the healthcare system is a serious challenge worldwide. This issue has long been a top concern for medical professionals throughout history, as mentioned in Hippocrates’ famous oath and his ethical writings on law and honesty [49]. Even Galen condemned medical quackery in his writings [50]. Fraudsters and charlatans lack the necessary scientific qualifications, and their actions, driven by greed, often result in detrimental consequences that weaken the entire health system.

Through our research, we have identified six that main causes of quackery in the health system: political, economic, sociocultural, technical-organizational, legal, and psychological. Additionally, the consequences of these fraudulent practices can be divided into three areas: social, economic, and health.

Of particular importance are the economic causes and economic consequences of health care fraud. Poor economic status of the society, increasing the cost of standard health services and insufficient supply of legitimate drugs push forcefully people to unverified products and services whose validity and quality haven’t been confirmed but they are affordable for them [20, 22, 23]. The phenomenon of “disease mongering” by providers and patients’ use of counterfeit services and products not only depletes the limited financial resources of patients but also puts their health at risk [24, 28]. Our findings align with those of the Andriote’s study on economic and health consequences of disease mongering [51]. In order to compensate for the consequences of receiving fraudulent services, the healthcare system must resort to expensive medical procedures that impose significant financial burdens [30, 39]. Stowell’s research also highlighted the high economic costs of healthcare fraud and its damaging impact on the public trust in the healthcare system [52].

Social factors also play a crucial role in the spread of quackery. When people have insufficient information and health literacy and are not knowledgeable enough to distinguish between standard and unauthorized services, they easily fall prey to deceptive advertisements on social media and in society. This leads them to use counterfeit products and services, resulting in harmful consequences [20, 30, 31]. As a result, their trust in legitimate healthcare services and products may diminish [20, 46]. This aligns with the results of Shao’s study et al. on the role of knowledge in the spread of medical quackery [53]. Furthermore, as quackery becomes more prevalent in the field of health, people may become distrustful of the entire health system and its professionals and they refuse to see them, ultimately leading to a decline in the overall health of the society [42, 46, 47]. The results of the Kovacs’ study et al. also supports this notion, highlighting the negative impact of fake and substandard drugs on public trust in health professionals [54].

The occurrence of quackery in the healthcare system is significantly influenced by legal causes. Insufficient laws and regulations, coupled with non-deterrent criminal penalties for profiteers, encourage quacks to continue their unethical and unauthorized activities more boldly, ultimately endangering the lives of patients [20, 21, 32]. The results of Wertheimer’s study et al. also highlights the impact of corruption on the effective implementation of laws [55]. Additionally, Li and Yang’s study emphasizes the vulnerability of China’s food production industry to corruption and strongly recommends the improvement of laws, policies and strict punishments [56].

Technical-organizational factors play a significant role in the spread of quackery in the healthcare system. The rapid growth and lax oversight of online pharmacies, as well as the lack of coordination among stakeholders in health system when it comes to preventing, detecting and countering quack medicine, contribute to the expansion of this problem [20, 24]. The findings of Thahab’s study reveal of the negative economic and health impacts of fake online pharmacies on individuals, organizations, companies and governments, emphasizing the need for legal measures and the cooperation among interested institutions [57]. Similarly, Al-Shahrani et al. listed the lack of cooperation among stakeholders as a major obstacle to implementing detection and monitoring technologies for counterfeiting in Saudi Arabia’s pharmaceutical industry [58]. The health consequences of quack are significant, and range from ineffective treatment to mortality [22, 24, 39]. Nicholas study et al. demonstrates a link between receiving medical care from fraudulent providers and increased mortality and emergency hospitalization [59].

### Strengths and limitations

The present study is one of the first studies to comprehensively investigate the causes and consequences of quackery in the healthcare system. By addressing the various dimensions of the issue, it provides a clear understanding of the importance of the problem for stakeholders. In fact, previous studies have focused on explaining the economic, legal and health problems and challenges of quackery in the health sporadically, while the present research, in addition to these dimensions, investigates the technical-organizational, political, sociocultural, psychological causes as well as social effects of quackery in health system. However, this study has limitations. Non-English studies related to the topic were not included due to the set inclusion and exclusion criteria. Future studies could explore different models and frameworks to categorize the findings. Additionally, the focus of this study is on the problem of quack medicine in the health service provision, and does not consider its impacts on education and research areas of the health system, which could be further investigated in future studies. Another limitation is related to the methodology of the study. While scoping reviews are valuable tools for mapping the existing literature on a specific topic, they also have certain drawbacks and limitations. For example, the depth of analysis in scoping review is shallow, the quality assessment of studies is not as strong and strict as systematic reviews, and also they suffer from potential bias in study selection and difficulty in managing heterogeneity.

## Conclusion

Based on the results of this research, the role of economic and social causes and consequences in relation to medical quackery is more prominent than other categories. Legal and technical-organizational factors were also found to play an important role in the spread of quackery. Furthermore, the health consequences of this issue can lead to irreparable damages. The results of this study highlight the importance of a comprehensive understanding of the causes and consequences of charlatanism in the healthcare sector for policy-makers and planners to adopt all-embracing and effective solutions to deal with this issue. It is recommended that the healthcare systems, prioritize addressing economic and sociocultural factors in order to effectively combat this issue. In developing solutions, attention must be given to cultural development and community education, and efforts should be made to strengthen mechanisms that provide access to affordable, standard healthcare services for all. Lastly, it is crucial to enhance the performance of systems responsible for legislation, implementation and evaluation of laws and regulations related to quack medicine.

## Data Availability

All datasets analyzed during the current study are available from the corresponding author on reasonable request.

## Appendix 1: Search strategy of databases

“quack medicine” **OR** “fake medicine” **OR** “Counterfeit Drugs” **OR** “Counterfeit Medicine” **OR** “Falsified Drugs” **OR** “medical quackery” **OR** “medical fraud” **OR** “health fraud” **OR** “medical scam” **OR** “medical deception” **OR** “medical swindle” **OR** “medical malversation” **OR** “medical trickery” **OR** “medical corruption” **OR** “Disease mongering” **OR** trickery **OR** misrepresentation **OR** deceit **OR** fraud **OR** Deception **OR** skullduggery **OR** Swindle **OR** malversation **OR** fake **OR** fakery **OR** counterfeit **OR** corruption **OR** malpractice **OR** quack **OR** quackery **OR** dishonesty **OR** cheat **OR** malfeasance **OR** mountebank **OR** jobbery **OR** profiteer **OR** scam **OR** charlatan **OR** charlatanism **OR** monger **OR** forgery **OR** misconduct **OR** falsification **AND** Health **OR** medicine **OR** medical **OR** therapy **OR** therapeutic **OR** cure **OR** heal **OR** treatment.

## Abbreviations

PRISMA: Preferred Reporting Items for Systematic Reviews and Meta-Analyses
WHO: World Health Organization
ADR: Adverse Drug Reactions
